# Curcumin: Biological Activities and Modern Pharmaceutical Forms

**DOI:** 10.3390/antibiotics11020135

**Published:** 2022-01-20

**Authors:** Maja Urošević, Ljubiša Nikolić, Ivana Gajić, Vesna Nikolić, Ana Dinić, Vojkan Miljković

**Affiliations:** Faculty of Technology, University of Niš, Bulevar Oslobođenja 124, 16000 Leskovac, Serbia; nljubisa@tf.ni.ac.rs (L.N.); ivana@tf.ni.ac.rs (I.G.); nikolicvesna@tf.ni.ac.rs (V.N.); vojkan@tf.ni.ac.rs (V.M.)

**Keywords:** curcumin, metabolism, bioavailability, formulations, pharmacological activities

## Abstract

Curcumin (1,7-bis-(4-hydroxy-3-methoxyphenyl)-hepta-1,6-diene-3,5-dione) is a natural lipophilic polyphenol that exhibits significant pharmacological effects in vitro and in vivo through various mechanisms of action. Numerous studies have identified and characterised the pharmacokinetic, pharmacodynamic, and clinical properties of curcumin. Curcumin has an anti-inflammatory, antioxidative, antinociceptive, antiparasitic, antimalarial effect, and it is used as a wound-healing agent. However, poor curcumin absorption in the small intestine, fast metabolism, and fast systemic elimination cause poor bioavailability of curcumin in human beings. In order to overcome these problems, a number of curcumin formulations have been developed. The aim of this paper is to provide an overview of recent research in biological and pharmaceutical aspects of curcumin, methods of sample preparation for its isolation (Soxhlet extraction, ultrasound extraction, pressurised fluid extraction, microwave extraction, enzyme-assisted aided extraction), analytical methods (FTIR, NIR, FT-Raman, UV-VIS, NMR, XRD, DSC, TLC, HPLC, HPTLC, LC-MS, UPLC/Q-TOF-MS) for identification and quantification of curcumin in different matrices, and different techniques for developing formulations. The optimal sample preparation and use of an appropriate analytical method will significantly improve the evaluation of formulations and the biological activity of curcumin.

## 1. Introduction

The main ingredient of the *Curcuma longa* is the rhizome [1], a low-molecular-weight lipophilic molecule that can pass through the cellular membrane easily [2]. By its chemical structure, it belongs to the group of polyphenols [3]. Because of its intensive yellow colour, it is used as a natural food colouring agent [1]. The simple molecular structure and arrangement of functional groups are suitable for examining the relationship between the structure and its activity [4]. The ability of curcumin to interact with different proteins facilitates selective modulation of multiple cellular signalling pathways associated with various chronic diseases [5]. The transcription factors, mediators of inflammation and enzymes such as protein kinase, reductase, and histone acetyltransferase are important molecules for curcumin binding. Curcumin is a powerful epigenetic regulator in many diseases, such as neurological disorders, inflammatory diseases, diabetes, and different types of cancer [6]. Furthermore, it modulates various proteasomal pathways and reduces glycogen metabolism through selective inhibition of phosphorylase kinase enzyme [7]. The studies have shown that curcumin exhibits anti-inflammatory, hypoglycemic, antioxidant, antimicrobial, antiviral, anticancer, neuroprotective, and many other effects [8]. However, the main obstacle to the effective manifestation of the pharmacological activity of curcumin is its poor aqueous solubility and low bioavailability [9,10,11]. The main factors contributing to the low bioavailability of curcumin in the blood plasma and tissues are its poor absorption, fast metabolism, and rapid systemic elimination [12]. The enhancement of the solubility and bioavailability of this promising molecule is crucial for potential clinical application. Different approaches in developing curcumin formulations can improve its physicochemical characteristics and enable safe and efficient use. For that purpose, formulations including nanoparticles, liposomes, micelles, phospholipid complexes, hydrogels, etc., have been described in the reference sources [11,13]. The aim of this paper is to analyse the factors influencing the bioavailability of curcumin, as well as to review pharmacological activities of curcumin and strategies in order to enhance its bioavailability.

## 2. Curcumin: Background

**Figure 1:**
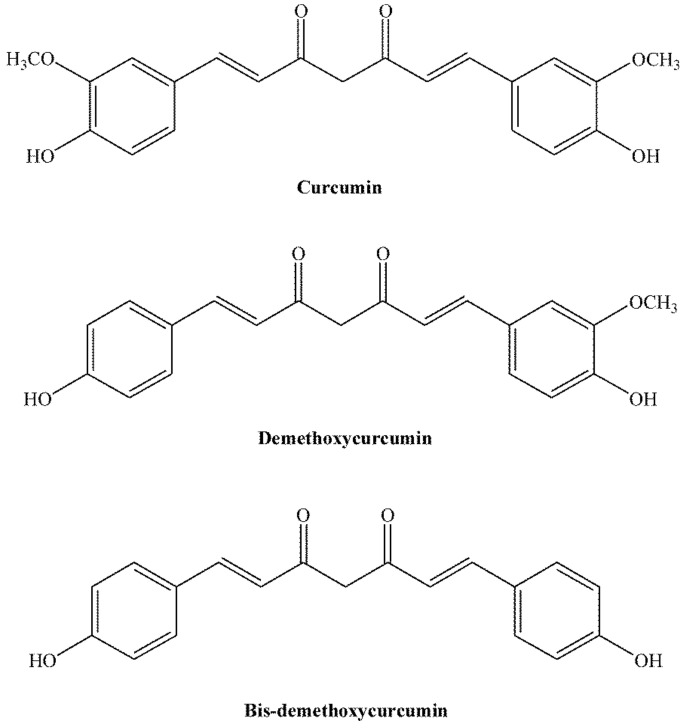
Structural formulae of curcuminoids.

Turmeric (*Curcuma longa*) is an aromatic plant from the ginger family (Zingiberaceae). It is grown in the southern and southwestern regions of Asia. It occupies an important place in the cuisine of Iran, Malaysia, India, China, Polynesia, and Thailand. It is used as a spice, and it affects the nature, colour, and taste of food. Curry is the best-known spice that contains turmeric rhizome powder. Curcumin is also used as an ecological dye; it is known as Natural yellow 3 and has been assigned an E number-E100, when used as a food colouring agent [14,15]. Figure 1 shows curcuminoids (curcumin, demethoxycurcumin, and bis-demethoxycurcumin), the main components of turmeric rhizome.

Curcumin, a yellow-orange pigment isolated from turmeric two centuries ago, is a widely studied natural compound that has shown enormous in vitro therapeutic potential. For centuries, it has been used in Ayurvedic medicine and traditional Chinese medicine [11,16]. An overview of the discovery and application of curcumin is shown in Table 1.

Curcumin has been found to possess pleiotropic activities owing to the potential of this polyphenol to modulate multiple signalling molecules. Curcumin exhibits anti-inflammatory, antioxidant, proapoptotic, chemopreventive, chemotherapeutic, antinociceptive, antiproliferative, antiparasitic, and antimalarial effects, and it is used as a wound-healing agent. The research on curcumin and its pharmacological activities has become increasingly important in recent years [9].

## 3. Isolation of Curcumin from Turmeric Rhizome and Methods of Identification

Turmeric rhizome contains two main classes of pharmacologically active secondary metabolites: curcuminoids and essential oil [28]. Curcuminoids (curcumin, demethoxycurcumin and bis-demethoxycurcumin) are most responsible for the biological activity of turmeric [29]. The isolation of curcuminoids from turmeric rhizomes is achieved by applying conventional and modern extraction methods [30]. Soxhlet extraction [31] and maceration [32] are classic extraction methods. Of the modern methods for extraction of curcuminoids, ultrasound extraction [33], enzyme-assisted extraction [34], microwave extraction [35], supercritical fluid extraction [36], and pressurized fluid extraction [37] are used. The most commonly used solvents for curcuminoid extraction are ethanol, dichloromethane, ethyl acetate, isopropanol, methanol, n-butanol, and acetone [29,38,39]. Sahne et al. used acetone as a solvent in conventional and unconventional extraction processes due to its high solubilization capacity [31]. In the paper by Muthukumar et al., various organic solvents for curcumin extraction were examined. The research findings show that acetone is the most efficient extraction solvent [39]. Thin-layer chromatography (TLC) is a classical analytical technique for separating curcumin from the extraction mixture [29,38]. Curcumin is quantified in the extract using high-performance liquid chromatography (HPLC). After extraction, the organic solvents are removed from the extract by evaporation on a vacuum evaporator. The residue (oleoresin) is then dissolved in methanol and subjected to HPLC analysis [40]. The yield and stability of curcumin depend on the extraction method used. Sahne et al. analysed curcumin extraction from the turmeric rhizome using several advanced methods, and the results were compared with the results obtained by Soxhlet extraction, the most commonly used reference method. The result showed that the yield of curcumin extraction obtained using the Soxhlet method (6.9%) was significantly higher than the one obtained by extraction using microwaves (3.72%), ultrasound (3.92%), and enzymes (4.1%). Although modern extraction methods do not show high extraction yields similar to the Soxhlet method, their advantages (low temperature and short extraction time, use of a very small amount of solvent) make them more favourable methods for curcumin extraction [31].

The kinetic degradation of curcumin from a natural mixture of curcuminoids in different conditions (pH, temperature, and dielectric constant of the solvent), as well as the degradation of pure curcumin in defined conditions, were examined in the paper by Naksuriya et al. An aqueous buffer/methanol 50:50 (*v*/*v*) mixture was used as a standard medium to examine the kinetics of curcumin degradation. The results showed that the degradation of pure curcumin present in the curcuminoid mixture underwent a first-order reaction. An increase in pH, temperature, and dielectric constant of the medium lead to an increase in the rate of curcumin degradation. Curcumin showed rapid degradation due to autoxidation in aqueous buffer pH = 8.0 with a constant rate of 0.28 h^−1^, which corresponds to a half-life (t_1/2_) of 2.5 h. Curcumin incorporated as a mixture of curcuminoids into ω-methoxypoly(ethylene glycol)-*b*-(*N*-(2-benzoyloxypropyl) methacrylamide) polymer micelles was about 300–500 times more stable than pure curcumin in a mixture of phosphate buffer and methanol.

Incorporating curcumin into polymer micelles is a promising approach for stabilising this compound and developing formulations suitable for further pharmaceutical and clinical trials [41]. Liu et al. examined natural deep eutectic solvents formed from organic acids and sugars for the efficiency of curcuminoid extraction. In optimal conditions (the temperature of 50 °C, 0.1/10 g/mL ratio of solid and liquid components, and extraction time of 30 min), higher extraction yields were achieved when a solvent with a ratio of citric acid and glucose 1:1 and 15% water was used, compared to the conventional extraction solvents. The proposed method is an excellent alternative for extracting natural pigments since it is environmentally friendly and sustainable [42]. During the isolation and purification of curcuminoids from oleoresin, the volatile oil of turmeric dissolves curcumin, thus creating a problem in the recrystallization process. Different organic solvents and their combinations for selective recrystallization of curcuminoids were examined. A mixture of isopropyl alcohol and hexane (1:1.5, *v*/*v*) proved to be the best solvent for recrystallization in the purification of curcuminoids. The total curcumin content in the raw curcuminoid powder was 76.82% *w*/*w*, whereas, in the recrystallized powder, the purity was increased up to 99.45% *w*/*w* [29].

## 4. Physico-Chemical Properties of Curcumin

**Figure 2:**
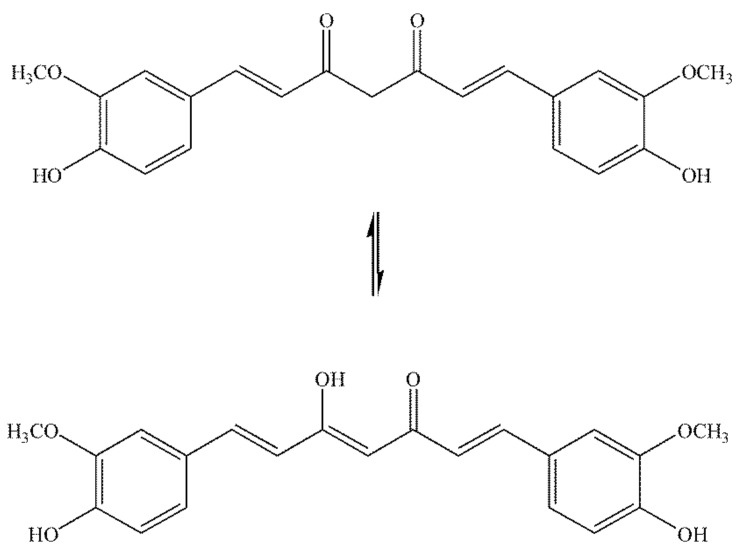
Keto-enol tautomerism of curcumin.

Curcumin (1,7-bis-(4-hydroxy-3-methoxyphenyl)-1,6-heptadiene-3,5-dione) [43] or diferuloylmethane [44] is an integral component of turmeric (up to ~5%), a well-known traditional spice [45]. It is a lipophilic compound, insoluble in water, acidic, and neutral solutions, and soluble in ethanol, dimethylsulfoxide, and acetone. Curcumin can be extracted from turmeric rhizomes by using organic solvents. The molecular formula of curcumin is C_21_H_20_O_6_, and the molecular weight is 368.38 g/mol. The melting point of curcumin is 183 °C. Curcumin is a tautomeric compound due to the presence of β-diketone in the molecular structure and shows diketo/keto–enol tautomerism (Figure 2) [46,47].

The diketo tautomer can exist in *cis* and *trans* forms. Solvent polarity, pH, and temperature significantly affect curcumin’s keto–enol balance [48]. The ratio of keto and enol tautomers of curcumin, on the other hand, strongly influences pharmacological activities [49]. Manolova et al. examined the tautomerism of curcumin in ethanol/water binary mixtures using ultraviolet–visible (UV–VIS) spectroscopy and advanced quantum chemical calculations. The results show that only enol–keto tautomer is present in ethanol. The addition of water leads to the emergence of a new spectral range, which is assigned to the diketo tautomeric form. The diketo form is dominant in the mixture of water and ethanol 90:10 (*v*/*v*). The observed equilibrium shift is explained by quantum chemical calculations, which show that water molecules stabilise the diketo tautomer by forming stable complexes [50]. Kawano et al. analysed keto–enol tautomers of curcumin by using liquid chromatography/mass spectrometry. The research findings show that the enol form is the main form in solution (water/acetonitrile) [51]. In nonpolar solvents (carbon tetrachloride) in the solid state and solution, curcumin exists in enol form [50,51].

Curcumin is unstable in the solution form. It has an intense yellow colour, which changes to dark red in the basic solution [52].

## 5. Structure, Bioavailability, and Safety of the Application of Curcumin

**Figure 3:**
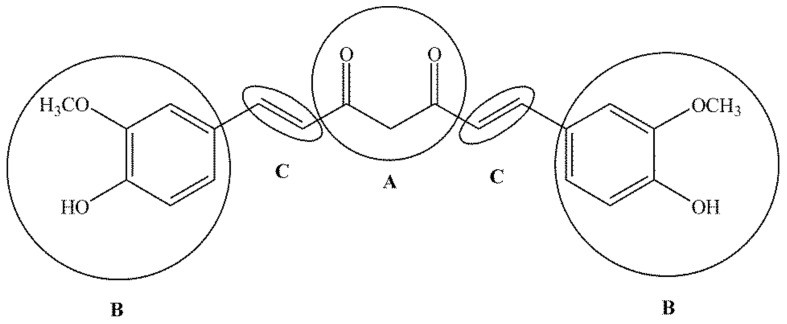
Important functional parts of curcumin: 1,3-keto-enol part (**A**), *o*-methoxy and phenolic groups (**B**) and a double bond (**C**).

Curcumin, a polyphenol from the diarylheptanoid group, has two aromatic rings symmetrically substituted by methoxy and a phenolic OH group in the *ortho* position, which are connected to a conjugated seven-membered hydrocarbon chain with an enone part and a 1,3-diketone group (Figure 3). The active functional groups of curcumin are two *o*-methoxy and two phenolic groups, two double bonds in the hydrocarbon chain and the 1,3-keto–enol part of the structure [53].

Aromatic groups provide hydrophobicity, whereas the α,β-unsaturated β-diketo part of the structure allows flexibility to the molecule. These unique properties of curcumin make it capable of binding to various biomacromolecules. Biologically critical chemical reactions of curcumin are realized through the H-bond of the β-dicarbonyl group and phenolic hydroxyl residues, as well as the ether residue of the methoxy group, and by binding with metals and nonmetals. It has been demonstrated that curcumin binds directly to numerous signalling molecules, such as inflammatory molecules, protein kinase, protein reductase, histone acetyltransferase, histone deacetylase, glyoxalase I, xanthine oxidase, human immunodeficiency virus (HIV1) integrase, HIV1 protease, sarco/endoplasmic reticulum calcium ATPase, deoxyribonucleic acid (DNA) methyltransferase 1, carrier proteins, and metal ions. The diketo group forms chelates with transition metals, reducing metal-induced toxicity, while some of the metal complexes exhibit enhanced antioxidant activity because they mimic enzymes [54]. Curcumin can also bind directly to DNA and ribonucleic acid (RNA). The ability of curcumin to bind to carrier proteins improves its solubility and bioavailability. Curcumin is unstable at physiological pH and degrades rapidly in an autoxidation reaction to the major bicyclopentadione product in which a 7-carbon chain has undergone oxygenation and double cyclization [55]. The alkaline hydrolysis products (ferulic acid, vanillin, ferulaldehyde, and feruloylmethane), as well as oxidation products (such as bicyclopentadione), show biological activity but are significantly less active than curcumin [56].

The clinical trials with curcumin have clearly demonstrated its safety, tolerability, and efficacy against different chronic diseases in humans [8]. The human studies did not indicate any toxic effects when curcumin was administered orally in the dosage of 6 g/day during 4–7 weeks [57]. The study on safety, tolerability, and activity of liposomal curcumin (Lipocurc™) on patients with locally advanced or metastatic cancer was conducted by Greil et al. It demonstrated that 300 mg/m^2^ of liposomal curcumin was the maximum safe dosage for patients with cancer treatment [58]. Saghatelyan et al. assessed the efficacy and safety of intravenous infusion of curcumin in combination with paclitaxel in patients with metastatic and advanced breast cancer. After a 12-week treatment, curcumin administered intravenously did not cause any significant health issues, nor did it deteriorate the quality of life [59].

## 6. The Metabolism of Curcumin

**Figure 4:**
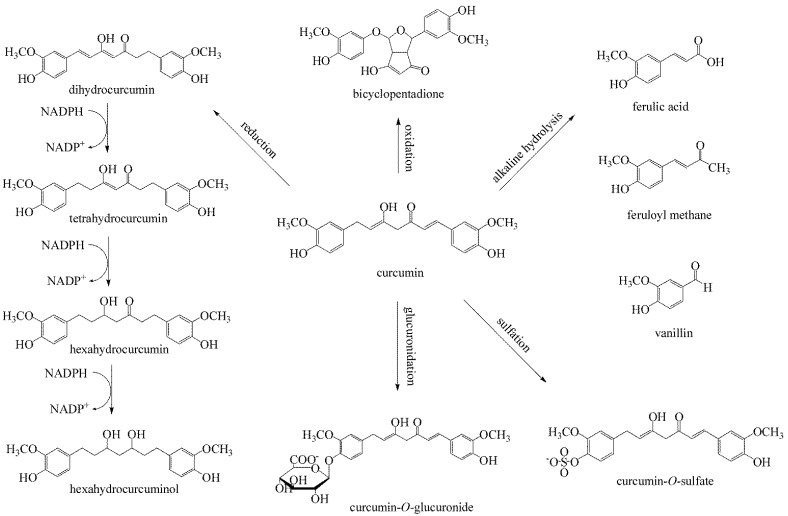
Important metabolic and nonmetabolic transformations of curcumin.

Poor bioavailability of curcumin in humans at a dose of 12 g/day is a consequence of poor absorption in the small intestine, fast metabolism in the liver, and rapid systemic elimination [60]. Most of the orally administered curcumin is excreted through faeces, without metabolism, while a smaller, absorbed part undergoes metabolic modification. The metabolism of curcumin takes place in two stages. The first phase involves the reduction in the presence of reductases, which takes place in enterocytes and hepatocytes. The reduction products are dihydrocurcumin, tetrahydrocurcumin, hexahydrocurcumin, and octahydrocurcumin (hexahydrocurcuminol) [61]. The curcumin reduction reaction is catalyzed by enzymes such as nicotinamide adenine dinucleotide phosphate (NADPH)-dependent reductase, alcohol dehydrogenase, and an unidentified microsomal enzyme [62]. In the paper by Hassaninasab et al., the enzyme for curcumin reduction was purified from *Escherichia coli* and characterized. It was found that the microbial metabolism of curcumin by a purified enzyme involved reduction in two steps, in which, depending on NADPH, curcumin was converted into an intermediate product, dihydrocurcumin, and then into the final product, tetrahydrocurcumin [63]. Curcumin and its reduced metabolites are readily conjugated to glucuronic acid and sulfate in vivo and in vitro. Glucuronidation and sulfation reactions take place in the presence of glucuronyl transferase and sulfotransferase, respectively. Glucuronidation and sulfation of curcumin occur in the liver and intestines of rats and humans [61]. After oral administration in humans, a portion of curcumin is absorbed and can be identified as a water-soluble glucuronide and sulfate conjugate in the plasma. Human phenol sulfotransferase 1A1 (SULT1A1) and human phenol sulfotransferase 1A3 (SULT1A3) are responsible for the sulfation of curcumin in humans and in the intestines of rats, while uridine diphosphate-glucuronosyltransferase (UGT) catalyses glucuronidation of curcumin [54]. The reduction or conjugation of curcumin generates species with a reduced ability to inhibit the expression of cyclooxygenase-2 (COX-2) compared with curcumin. Tetrahydrocurcumin, hexahydrocurcumin, and curcumin sulfate exhibit weaker inhibition of prostaglandin E2, while hexahydrocurcuminol is inactive [64]. The biological activity of curcumin metabolites other than tetrahydrocurcumin is significantly reduced compared with curcumin [65,66]. To enhance the bioavailability of curcumin, piperine which interferes with glucuronidation, curcumin in liposomes, curcumin nanoparticles, curcumin phospholipid complexes, and structural curcumin analogues are used. Figure 4 shows the metabolic and nonmetabolic transformations of curcumin.

## 7. Characterization of Curcumin

Curcuminoids are widely used in the food processing and pharmaceutical industries due to their properties. The detection and characterization of curcuminoids in different matrices are of great importance. The choice of the analytical method for curcuminoid analysis depends on the sample type, the purpose of the analysis, and the detection and quantification limits [67,68]. The techniques based on chromatography and electrophoresis are among the selected methods for determining curcuminoids. TLC is one of the methods used for fractioning turmeric extracts [38]. The use of the TLC method for turmeric analysis has declined due to prolonged separation time and poor resolution, although it is selective, easy to perform, and inexpensive. New high-performance thin-layer chromatography (HPTLC) methods that overcome these limitations have been developed [69]. The principle of operation is the same as with TLC. Higher resolution, lower detection limit and improved image scanning are advantages of HPTLC methods [68]. HPLC, in combination with UV–VIS detector, is the most commonly used chromatographic method for the qualitative and quantitative analysis of curcumin due to its high precision, accuracy, and low detection limit. Various HPLC methods have been developed to analyse curcuminoids (Table 2).

Liquid chromatography–mass spectrometry (LC/MS) [67] is used to identify and quantify traces of curcumin in biological fluids, food, or other complex matrices. Various LC/MS methods have been developed to detect and quantify curcumin in different matrices [77,78,79]. A rapid and sensitive, selective high-throughput ultrahigh performance liquid chromatography method with tandem mass spectrometry (UPLC/Q-TOF-MS) was developed and validated to quantify curcuminoids to reduce analysis time and improve sensitivity [80]. The UV–VIS spectroscopy can also quantify curcuminoids if the sample matrix or other present components do not absorb within this range. Curcumin shows an absorption maximum at 425 nm [62,68,81]. Fourier transform infrared spectroscopy (FTIR), near-infrared spectroscopy (NIR), Raman’s spectroscopy, nuclear magnetic resonance spectroscopy (NMR), and fluorescence spectroscopy are also used to characterize curcumin [82,83,84,85].

Curcumin exists in three polymorphic forms: monoclinic form and two orthorhombic forms. Pandey et al. examined polymorphs using X-ray diffraction (XRD) and differential scanning calorimetry (DSC) and found that curcumin polymorphs were monotropically linked to each other, with the monoclinic form being the most stable [86].

Electron paramagnetic resonance (EPR) spectroscopy is an efficient and noninvasive spectroscopic method for analysing samples with unpaired electrons. It is used to quantify the types of radicals and analyse the antioxidative effects of substances [87]. EPR spectroscopy was applied for determining the potential and capacity of curcumin against free radicals (DPPH, nitric oxide radical (NO⋅), hydroxyl radical (HO∙) and superoxide anion radical (O_2_) [88,89]. In the study by Nikolić et al., EPR spectroscopy was used for assessing the antioxidant activity of curcumin-loaded low-energy nanoemulsions according to Tempol stable nitroxide free radical. The research findings show that nanoemulsion with curcumin exhibits swift activity, thus neutralising free radicals within the first five minutes from the beginning of the reaction [90].

## 8. Formulations

**Figure 5:**
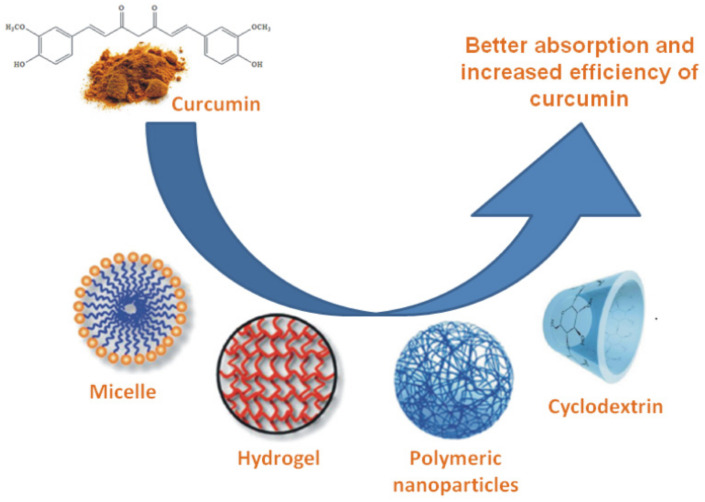
Formulations of curcumin.

A large number of the curcumin formulations with volatile oil (volatile oil formulation) [91,92], piperine [93], and lecithin [94] have been designed. These formulations increase the absorption of curcumin after oral administration compared with pure curcumin. Liposomes, micelles, phospholipid complexes, cyclodextrins, nanoparticles, emulsions, hydrogels, and phytosomes are new promising curcumin formulations. Such formulations provide more prolonged circulation, better absorption and resistance to metabolic processes, increase absorption from the small intestine, and prolong half-life in the plasma, and thus, increase the efficiency of curcumin [95,96,97] (Figure 5). 

Phytosome formulations with curcumin, formulations with volatile oils of turmeric rhizome, and curcumin formulations with a combination of hydrophilic carrier, cellulose derivatives, and natural antioxidants (CHC), compared to a standardized mixture of curcumin (CS), were tested in a study on healthy volunteers. The CHC formulation of curcumin significantly increases the content of curcuminoids in the blood compared with standard curcumin [92]. Cyclodextrins (CDs) can form molecular inclusion complexes with lipophilic compounds, thus enhancing water solubility, dispersion, and absorption of active components. The bioavailability of the curcumin formulation with γ-cyclodextrin was investigated. This formulation was compared with a standardized curcumin extract and two commercially available formulations, the curcumin phytosome formulation (CSL) and the curcumin formulation with rhizome-extracted turmeric essential oils (CEO). The formulation of curcumin with γ-cyclodextrin significantly enhances the absorption of curcuminoids in healthy people [9]. The inclusion complex of curcumin with β-cyclodextrin was prepared using the coprecipitation method. The solubility of curcumin in water increased from 0.00122 to 0.721 mg/mL by forming an inclusion complex. The release of the inclusion complex from nanocomposite and conventional poly (*N*-isopropylacrylamide/sodium alginate) hydrogels cross-linked with nanoclay and *N*,*N′*-methylenebis(acrylamide) (BIS), respectively, was tested under simulated gastrointestinal conditions. At pH = 1.2 and pH = 6.8, hydrogels showed the lowest and the highest release-swelling ratio, respectively. The swelling coefficient and cumulative release decreased with increasing nanoclay content in nanocomposite hydrogels. Conversely, as the BIS ratio in conventional hydrogels increased, the swelling ratio and cumulative release increased [98]. The polyol dilution method was used to formulate liposomes with curcumin in the paper by Kongkaneramit et al. Lipid phase was a mixture of hydrogenated phosphatidylcholine and cholesterol in a molar ratio of 9:1. Propylene glycol, glycerin, and polyethylene glycol 400 were used as polyol solvents. The type and amount of polyol affect both the size of the liposomes and the amount of curcumin incorporated. The preparation temperature is also an important factor in liposome production [99]. Tai et al. studied the stability and release performance of curcumin from liposomes with different contents of hydrogenated phospholipids [100]. 

Chitosan-coated liposomes may be an alternative carrier for drug delivery in humans. In the work of Cuomo et al., the applicability of chitosan-coated liposomes with curcumin, as well as anionic liposomes with curcumin, was evaluated. The applicability of the formulations was examined in vitro by measuring the bioavailability of ingested curcumin. It has been shown that the presence of a positively charged liposome surface enables better absorption of curcumin in the small intestine, which increases its overall bioavailability [101]. Curcumin nanoemulsion was formulated as a low-energy emulsion and converted to a nanoemulgel using cross-linked polyacrylic acid (Carbopol^^®^^ 934) as a gelling agent to increase the solubility and absorption of curcumin after topical application to the skin. The nanoemulgel formulation showed faster and earlier wound healing in psoriatic mice compared with curcumin and betamethasone-17-valerate gel. The research findings show that curcumin nanoemugel formulation is a promising candidate for successful long-term treatment of psoriasis [102].

Curcumin nanoemulsions are highly effective in preventing tumour recurrence after surgery and metastasis [103]. A formulation of eye drops (thermosensitive hydrogel containing latanoprost and curcumin nanoparticles) for dual drug delivery has been developed and characterized. The developed dual drug delivery system has shown a prolonged release profile, in vitro and in vivo biocompatibility, reduced levels of inflammation and apoptosis of cells, and protection of trabecular mesh (TM) cells from oxidative damage [104]. PLGA curcumin nanoparticles have shown increased oral and intravenous bioavailability [105]. The oral formulation of nanocurcumin can significantly reduce recovery time in hospitalized patients with COVID-19 [106]. The hybrid curcumin-phospholipid complex was used as a system for oral drug administration to inhibit the metastasis of breast and lung cancer [107]. A high-performance formulation of curcumin phospholipid complex, which can improve the flow, solubility, and oral bioavailability of curcumin, was developed by Wang et al. [108]. Polymer micelles made using block copolymer methoxy-poly(ethylene glycol) (mPEG)-poly(caprolactone) (PCL) enable delayed release of curcumin [109].

In the study by Karavasili et al., the activity of peptide hydrogel with simultaneous delivery of doxorubicin and curcumin in the therapy of head and neck cancer cells was examined. The findings showed the therapeutic utility of a double peptide hydrogel with built-in drugs for the local treatment of head and neck cancer [110]. The amylopectin–chitosan composite hydrogel (LRA–CS) for curcumin delivery was synthesized and tested by Liu et al. The release characteristics of encapsulated curcumin in the simulated gastric and intestinal fluid were observed. The findings showed that LRA–CS hydrogel provided stability of curcumin in the stomach and its release in the small intestine [111]. Chitosan–nanocellulose hydrogel with nonionic surfactant was also used for the delivery of curcumin [112]. Cyclodextrin nanospongoid-based hydrogel (CDNS) was used for transdermal codelivery of curcumin (CUR) and resveratrol (RES). Nanosponges enhanced the in vitro release of curcumin 10 times and the release of resveratrol 2.5 times compared with regular curcumin and resveratrol. The combination of CUR–CDNS and RES–CDNS demonstrated a synergistic cytotoxic effect on MCF-7 cells. A hydrogel base was developed with carbomer and propylene glycol, in which CUR-CDNS and RES-CDNS were incorporated. The photostability of curcumin and resveratrol in the CDNS hydrogel formulation increased almost five and seven times, respectively, compared with the hydrogel formulated without CDNS. Curcumin and resveratrol intake is significantly enhanced when delivered using a CDNS-hydrogel base [113]. In Shef et al., curcumin was incorporated into the oxidized cellulose–polyvinyl alcohol hydrogel system by the freezing process. In vitro studies on rats have shown that this can be an effective method for natural wound healing [114]. In the work of Sahin et al., a new, highly bioavailable formulation of curcumin, advanced ultrasol curcumin (AUC), with improved intestinal stability, was developed. In administered doses, AUC effectively improves the pathophysiology of osteoarthritis in experimentally induced osteoarthritis in rats [115]. An overview of curcumin formulations tested on humans and animals is shown in Table 3 and Table 4, respectively.

A study was conducted to compare the oral bioavailability of the newly developed formulation of curcumin Curene^®^ with a formulation of curcumin containing volatile turmeric oil (CP-01) and standard curcuminoids 95%, on healthy volunteers. It was found that the oral bioavailability of Curene^®^ is significantly higher compared with CP-01 and standard curcuminoids (95%) and that it is safe to be administered to healthy people in trial conditions [136]. The anti-inflammatory activity of Longvida^®^ Optimized Curcumin (LC) was examined on two-month-old wild-type mice and GFAP-IL6. LC can alleviate inflammation and minimize neurodegeneration and motoric defects in GFAP-IL6 mice [137]. There is a range of commercial formulations of curcumin with defined bioavailability and pharmacokinetics such as Meriva^®^, LongVida^®^, CurQfenTM, MicroActive curcumin, Micronized curcumin, NovaSol^®^ (micellar curcumin) CurcuWin^®^, BiocurcumaxTM Curcumin C3 Complex^®^+Bioperine, Cavacurmin^^®^^ Theracurmin^TM^. Of the commercial formulations, NovaSol^®^ (185), Curcuwin^®^ (136) and LongVida^®^ (100) stand out as they show bioavailability over 100 times higher than the reference curcumin [11]. The formulations CurcuminRich, Biomor, Liposomal curcumin mango, Liposomal curcumin, and Dr. Mercola Curcumin Advanced are also available on the market for oral administration of curcumin [95].

## 9. Biological Activities of Curcumin

**Figure 6:**
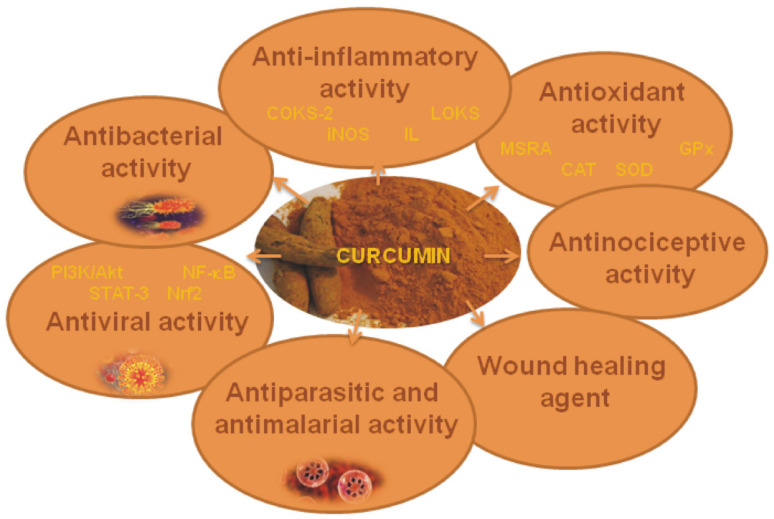
Biological activities of curcumin.

This section describes in detail the biological activities of curcumin, with a special emphasis on its antimicrobial activity (Figure 6).

### 9.1. Antimicrobial Activity

#### 9.1.1. Antibacterial Activity

Curcumin shows a wide range of antibacterial effects. It causes membrane damage in the cells of Gram-positive (*Staphylococcus aureus* and *Enterococcus faecalis*) and Gram-negative (*Escherichia coli* and *Pseudomonas aeruginosa*) bacteria [138]. It blocks the growth of bacteria owing to its structural characteristics and the formation of antioxidant products, inhibits bacterial virulence factors and the formation of bacterial biofilm, and prevents bacterial adhesion to host receptors. As a photosensitizer, curcumin induces phototoxicity and inhibits bacterial growth under blue light [139]. In the study by Adamczak et al., the effectiveness of curcumin was tested in vitro on over 100 strains of pathogens within 19 species. The antimicrobial activity was determined by the broth microdilution method and by calculating the minimum inhibitory concentration (MIC). The results confirmed a much higher susceptibility to Gram-positive than to Gram-negative bacteria. The MIC was also high in *Staphylococcus aureus*, *Staphylococcus haemolyticus*, *Escherichia coli*, and *Proteus mirabilis* resistant to a large number of drugs (≥2000 µg/mL). However, curcumin was effective against some species and strains: *Streptococcus pyogenes* (mean MIC = 31.25 µg/mL), *Staphylococcus aureus* sensitive to methicillin (250 µg/mL), *Acinetobacter lwoffii* (250 µg/mL) and single strains of *Enterococcus faecalis* and *Pseudomonas aeruginosa* (62.5 μg/mL). Curcumin shows poor activity against clinical isolates of *Candida species*. Curcumin can be considered a promising antibacterial agent but with very selective activity [140]. The antimicrobial activity of curcumin against pathogens in burn wounds is shown in Table 5.

#### 9.1.2. Antiviral Activity

The antiviral effect of curcumin is manifested through interference with virus replication or through suppression of cellular signalling pathways that are essential for virus replication, such as the phosphatidylinositol 3-kinase/protein kinase B (PI3K/Akt) and NF-κB [142]. Curcumin exhibits antiviral activity against DNA and RNA viruses [143]. Jeong et al. established a mechanism by which curcumin pretreatment controlled the early stage of viral haemorrhagic septicaemia virus (VHSV) infection in fathead minnow cells. By rearranging the F-actin/G-actin ratio through reduced regulation of heat shock cognate 71 (HSC71), virus entry into cells is suppressed [144]. Ferreira et al. found that curcumin significantly reduced the replication of HIV-1 and herpes simplex virus type 2 (HSV-2) in chronically infected T cells and human primary genital epithelial cells [145]. Curcumin is an inhibitor of the redox function apurinic/apyrimidinic endonuclease 1 (APE1), affecting many genes, thus accounting for the wide range of curcumin effects on various human diseases. Curcumin effectively blocks the replication of the herpes virus associated with sarcoma and inhibits the pathogenic processes of angiogenesis and cell invasion [146]. Curcumin also exhibits antiviral activity against Zika and chikungunya viruses, dengue virus, hepatitis C virus [147], coxsackievirus, human papilloma virus, severe acute respiratory syndrome coronavirus 2 (SARS-CoV-2), etc., [147,148,149].

SARS-CoV-2 is an infectious virus that causes coronavirus disease-2019 (COVID-19) [150]. The disease with significant mortality worldwide poses a global threat due to the difficulties in treatment because there is currently no approved antiviral drug with proven efficacy and minor adverse effects [151,152]. The severity of the pandemic has prompted scientists to examine existing drugs with the potential for treating COVID-19 [150]. Studies show that curcumin is a good candidate for treating the COVID-19 virus and preventing fatal complications of this disease due to its thoroughly tested and confirmed anti-inflammatory, antiviral, antinociceptive immunomodulatory, antipyretic, antifibrotic, pulmoprotective, and antifatigue effects. Curcumin can interact with spike proteins or angiotensin 2 (ACE2) proteins in the signalling pathway induced by COVID-19. Curcumin also inhibits several important signalling pathways in viral infection, such as transcription factors (NF-κB, signal transducer and activator of transcription 3 (STAT-3), Vnt/b-catenin, nuclear factor E2-related factor (Nrf2), p38/MAPK, and virus-induced inflammation by modulating the manifestation of various factors (IL-10, Interleukin-18 (IL-18), IL-6, tumour necrosis factor (TNF) α/β and COX-2) in COVID-19 [153,154]. Valizadeha et al. investigated the effect of nanocurcumin on the modulation of inflammatory cytokines in patients with COVID-19. Messenger ribonucleic acid (mRNA) expression and cytokine secretion levels of Interleukin-1 β (IL-1β), IL-6, TNF-α, and IL-18 were assessed by polymerase chain reaction (PCR) in real-time and enzyme-linked immunosorbent assay (ELISA), respectively. The results showed that the expression of IL-1β and IL-6 mRNA was dramatically reduced after nanocurcumin administration. This study suggests that by regulating the inflammatory response, nanocurcumin can be used as an innovative therapeutic agent for patients with COVID-19 [155].

#### 9.1.3. Antiparasitic and Antimalarial Activity

Curcumin shows activity against different types of parasites in vitro and in vivo. The antiprotozoal activity of curcumin *is shown* against *Leishmania major*, *Leishmania donovani* [156,157], *Trichomonas vaginalis* [158], *Entamoeba histolytica* [159], *Giardia lamblia* [160], *Toxoplasma gondii* [161], *Neospora caninum* [162], etc. The combinations of netilmicin and curcumin and metronidazole and curcumin show a synergistic effect and can be used to treat leishmaniasis and amoebiasis, respectively [156,159]. Curcumin also exhibits anthelmintic activity on the nematode *Ascaridia galli* and the cestode *Raillietina cesticillus* [163,164]. The studies show that curcumin is used to treat malaria and that it can increase the effectiveness of antimalarial drugs [165]. Busari et al. examined the in vivo antiplasmodial activity and the assessment of the toxicity of curcumin incorporated into poly(lactic-*co*-glycolic) nanoparticles. The formulated drug with nanoparticles demonstrated better activity against the malaria parasite than free curcumin. The antimalarial activity of the drug is better at lower concentrations. In vivo toxicity studies have confirmed the safety of the formulated drug at the tested doses [166]. Novaes et al. evaluated the efficacy of curcumin as a complementary strategy in benznidazole-based chemotherapy in mice acutely infected with *Trypanosoma cruzi*. The results of the research show that the combination of benznidazole with curcumin may be a relevant therapy in the treatment of Chagas’ disease caused by *T. cruzi* because it reduces the toxic effects of benznidazole and increases its antiparasitic activity [165]. An overview of the recent research of curcumin’s antiviral, antiparasitic, and antimalarial activities is shown in Table 6.

### 9.2. Anti-Inflammatory Activity

Reactive oxygen species (ROS) play a key role in enhancing inflammation by activating transcription factors NF-κB, the activator protein 1 (AP-1), in acetylation and deacetylation of nuclear histone in a range of inflammatory diseases [183]. The anti-inflammatory effect of curcumin is based on its ability to inhibit COX-2, lipoxygenase (LOX), inducible nitric oxide synthase (iNOS) [184], arachidonic acid metabolism [185], cytokines (interleukins) [186,187], and tumour necrosis factor [188], NF-κB [184] and the release of steroid hormones [189]. COX-2, LOX, and iNOS are important enzymes that mediate inflammatory processes. Improper regulation of COX-2 and iNOS has been associated with the pathophysiology of certain types of cancer in humans, as well as with inflammatory disorders [184]. Curcumin and rutin downregulate COX-2 and reduce tumour-associated inflammation in HPV16-transgenic mice [190] The findings of preclinical studies in animal models with invasive pneumonia showed that curcumin exhibits a protective effect. It regulates the expression of pro and anti-inflammatory factors (interleukin-6 (IL-6), interleukin-8 (IL-8), interleukin-10 (IL-10), and COX-2), induces apoptosis of polymorphonuclear neutrophilic (PMN) cells, and removes ROS, thus improving the inflammatory response. These studies indicate that curcumin can be used as a therapeutic agent against pneumonia and acute lung injury (ALI)/fatal acute respiratory distress syndrome (ARDS) in humans, resulting from coronavirus infection [191].

### 9.3. Antioxidant Activity

ROS and reactive nitrogen species (RNS) are generated in the human body in various endogenous systems, in pathophysiological conditions or exposure to various physical and chemical factors. Free radicals can change lipids (lipid peroxidation), proteins (loss of enzyme activity), and DNA (mutagenesis and carcinogenesis); they contribute to ageing and many human diseases. Natural protective antioxidant mechanisms include superoxide dismutase (SOD), catalase (CAT), glutathione (GSH), glutathione peroxidase (GPx), and reductase, vitamin E (tocopherols and tocotrienols), vitamin C, etc. [4]. Curcumin also shows strong antioxidant activity. The antioxidant property is attributed to the presence of various functional groups, including methoxy, phenoxy, and carbon–carbon double bonds in its structure. Curcumin is a classic phenolic antioxidant that donates H atoms from phenolic groups [192]. In the work of Samarghandian et al., it was found that curcumin can inhibit oxidative damage caused by stress in the brain, liver, and kidneys of rats [193]. Lipid peroxidation is significantly reduced in rats treated with curcumin before applying γ-radiation [194]. Curcumin increases enzymatic antioxidant activity by increasing the expression of methionine sulfoxide reductase (MSRA) and increasing the levels of the enzymes MSRA, SOD, CAT and GPx [195]. Curcumin may act as an antioxidant against oxidative stress in rats with diabetes mellitus by increased SOD expression in cochlear fibroblasts [196]. The antioxidant activity of curcumin was assessed using the 1,1-diphenyl-2-picryl hydrazyl (DPPH) radical assay compared to ascorbic acid, a known antioxidant. The percentage of free radical removal using curcumin and ascorbic acid was 69 and 62%, respectively, at a concentration of 0.1 mM. No significant difference was observed between curcumin and ascorbic acid in antioxidant potential [197]. Curcumin has shown a large capacity to remove smaller oxidative molecules such as H_2_O_2_, HO•, ROO•. Curcumin can be used as an effective antioxidant to protect against ROS in the cytoplasm of cells [198]. Curcumin formulations with different carriers that are stable and protected from various influences are used as antioxidants [199,200].

### 9.4. Antinociceptive Activity

Preclinical studies have shown that curcumin has an antinociceptive effect on inflammatory and neuropathic pain. The effects of curcumin on postoperative pain in rats were investigated in the work of Zhu et al. The results of the study show that curcumin can alleviate postoperative pain and accelerate recovery from surgery. However, treatment with curcumin before surgery did not affect the threshold of postoperative pain and recovery rate [201]. The antinociceptive effect of poly(d,l-lactide-*co*-glycolide) nanovesicles with curcumin (PLGA-CUR) administered intravenously or intrathecally in mice in small and high doses was tested using formalin test, zymosan-induced hyperalgesia and sciatic nerve ligation that causes neuropathic allodynia and hyperalgesia. PLGA-CUR administered intravenously managed to reduce the response to nociceptive stimuli in the formalin test and zymosan-induced hyperalgesia, while pure curcumin was inactive. The low doses of intrathecally administered PLGA-CUR significantly reduced allodynia produced by sciatic nerve ligation. Long-lasting antinociceptive effects were observed when high doses of PLGA-CUR were administered intrathecally. At high doses, intrathecally applied pure curcumin had only rapid and transient antinociceptive effects. Measuring cytokine levels and brain-derived neurotrophic factor (BDNF) in the spinal cord of neuropathic mice shows that the antinociceptive effects of PLGA-CUR depend on the decline in the release of cytokines and BDNF in the spinal cord. The study results show the efficacy of PLGA-CUR and suggest that the nanoformulation of PLGA-CUR could be a new potential drug in the treatment of pain [202].

### 9.5. Wound Healing Agent

Curcumin has strong modulating effects on the wound healing process. The wound healing process consists of four phases: coagulation, inflammation, proliferation, and tissue remodelling. Curcumin induces apoptosis of inflammatory cells during the early phase of wound healing, inhibits the activity of the transcription factor NF-κB, reduces the production of cytokines (TNF-α and IL-1), removes ROS, affects the production of antioxidant enzymes and thus reduces inflammation and shortens the inflammatory phase in the wound healing process. The studies have shown that during the proliferation phase, curcumin enhances fibroblast migration, enhances granulation tissue formation, collagen deposition, and re-epithelialization. In the final phase of wound healing, by increasing the production of the transforming growth factor β, curcumin enhances wound contractions and therefore increases fibroblast proliferation [203]. Various topical curcumin formulations such as films, fibres, emulsions, hydrogels, and various nanoformulations have been developed for targeted delivery of curcumin at the wounds [203,204,205]. Sodium alginate-g-poly(*N*-isopropylacrylamide), (Alg-pNIPAM), a thermosensitive hydrogel with incorporated curcumin as an in vivo wound dressing was synthesized by Zakerikhoob et al. In vivo studies have shown accelerated collagenesis, re-epithelialization, and wound contraction using the Alg-pNIPAM formulation with curcumin. The formulation showed a more significant anti-inflammatory effect than the free curcumin solution. Given the antioxidant and anti-inflammatory properties of curcumin and the concomitant effect of alginate in keeping wound areas moist, the developed thermosensitive formulation of curcumin could help accelerate wound healing [204]. An overview of the recent research about the biological activities of curcumin is shown in Table 7.

## 10. Conclusions

Curcumin is a widely studied natural compound that has exhibited enormous in vitro and in vivo therapeutic potential. Curcumin has anti-inflammatory, antioxidant, antiviral, proapoptotic, chemopreventive, chemotherapeutic, antinociceptive, antiproliferative, antiparasitic, and antimalarial effects and is used as a wound-healing agent. Poor absorption of curcumin in the small intestine, rapid metabolism, and rapid systemic elimination cause poor bioavailability of curcumin in humans. Different curcumin formulations are used for more prolonged circulation, better permeability, and resistance to metabolic processes, and thus to increase the efficacy of curcumin. Liposomes, micelles, phospholipid complexes, cyclodextrins, nanoparticles, emulsions, hydrogels, and phytosomes have been described in the reference sources as promising formulations that improve the physicochemical properties of curcumin and enable its safe and efficient use.

## Figures and Tables

**Table 1:** The History of curcumin.

Year	Discovery	Reference
1815	Vogel and Pelletier were the first to report the “Orange-yellow Substance” isolated from the rhizome of *Curcuma longa* and named it curcumin.	[16]
1842	Vogel Extracted pure preparation of curcumin but did not report its formula.	[17]
1910	Milobedzka and Lampe identified chemical structure of curcumin as diferuloylmethane, or 1,6-heptadiene-3,5-dione-1,7-bis-(4-hidroxy-3-methoxyphenyl)-(1E, 6E).	[18]
1913	The synthesis of curcumin was published.	[19]
1949	Schraufstatter et al. Reported that curcumin is a biologically active compound with antibacterial properties.	[20]
1953	Srinivasan separated and quantified the components of curcumin using chromatography.	[21]
1971	It was discovered that curcumin lowers cholesterol	[22]
1972	It was discovered that curcumin lowers the level of sugar in the blood	[23]
1973	It was discovered that curcumin has an anti-inflammatory effect	[24]
1976	It was discovered that curcumin has an antioxidant effect	[25]
1980	Kuttan et al. demonstrated anticancer activity of curcumin both in vitro and in vivo.	[26]
1995	Curcumin exhibits anti-inflammatory activity by suppressing the proinflammatory transcription factor, nuclear factor-kappa B (NF-κB)	[27]

**Table 2:** HPLC methods for curcuminoid analysis.

Matrix Sample	Column	Mobile Phase	λ, nm	Limit of Detection	Reference
Turmeric Powder	RP C_18_	Acetonitrile and 0.1% Trifluro-Acetic Acid (50:50, *v*/*v*)	420	27.99 ng/mL	[70]
Turmeric Extracts	Alltima C_18_ column	Acetonitrile and 2% Acetic Acid (40:60, *v*/*v*)	425	0.90 μg/mL	[71]
Commercial Samples of Turmeric	C_18_	Methanol, 2% Acetic acid, and Acetonitrile	425	0.05 µg/mL	[72]
Curcuminoids-Loaded Liposome	Zorbax Eclipse XDB C_18_ (4 × 150mm, 5 µm)	Acetonitrile and 0.1% OrthoPhosphoric Acid (50:50, *v*/*v*)	425	0.124 µg/mL	[73]
Samples of Turmeric	Zorbax SB-C_18_ column (4.6 × 250 mm, 5 µm)	Acetonitrile and 0.4% Aqueous Acetic Acid	430	0.31 μg/mL	[37]
Extract of Turmeric	C_18_ (4.6 × 150mm, 5 µm)	Acetonitrile and 2% Acetic Acid (55:45, *v*/*v*)	425	0.0738 ppm	[74]
Extract of Turmeric	Waters Xterra MS C_18_ column (4.6 × 250 mm, 5 µm)	Distilled Water and Acetonitrile (65:35, *v*/*v*) Containing 1% Acetic Acid	425	1.13 μg/mL	[75]
Turmeric Rhizome	Brownlee SPP C_18_ column (4.6 × 100 mm, 2.7 µm)	Water and Acetonitrile (70:30, *v*/*v*)	420	1.0 μg/mL	[76]

**Table 3:** Clinical applications of curcumin.

Disease	Dose	Duration	Patients	Results	Reference
Overweight	80 mg/Day	6 Weeks	48 Overweight Girl Students	Positive antioxidant effect and prevention of lipid peroxidation in overweight individuals.	[116]
Metabolic syndrome(MetS)	80 mg/Day	12 Weeks	50 Patients	Supplementation with Nanomicelle curcumin Significantly improved serum triglyceride in MetS patients.	[117]
Diabetic sensorimotor polyneuropathy	80 mg/Day	8 Weeks	80 Diabetic patients	Nanocurcumin supplementation reduced the severity of diabetic sensorimotor polyneuropathy in patients with type 2 diabetes mellitus.	[118]
Migraine	80 mg/Day	2 Months	80 Patients	Combination of omega-3 fatty acids and nanocurcumin modulates interleukin-6 gene Expression and high-sensitivity C-reactive protein serum levels in patients with migraine.	[119]
Nonalcoholic fatty liver disease (NAFLD)	80 mg/Day	3 Months	84 Patients	Nanocurcumin improves glucose indices, lipids, inflammation, and nesfatin in overweight and obese patients with nonalcoholic fatty liver disease (NAFLD).	[120]
Hemodialysis (HD)	120 mg/Day	12 Weeks	60 Patients	Nanocurcumin shows beneficial effects in lowering inflammation and Hs-CRP levels, as well as adhesion molecules (ICAM-1, VCAM-1), in hemodialysis patients.	[121]
Overweight and obesity	500 mg/Day	10 Weeks	60 Adolescent	Ten weeks of curcumin supplementation had beneficial effects on inflammation and oxidative stress markers among postpubescent overweight and obese girl adolescents.	[122]
Coronavirus disease-2019	1050 mg/Day	14 Days	158 Patients	Curcumin is a safe and natural therapeutic option to prevent post-COVID-19 thromboembolic events.	[123]
Ulcerative colitis (UC)	450 mg/Day	8 Weeks	41 Patients	Low-dose oral curcumin is not effective in inducing remission in mild-to-moderate ulcerative colitis.	[124]
Ulcerative colitis (UC)	1500 mg/Day	8 Weeks	70 Patients	Consumption of the curcumin supplement, along with drug therapy, significant improvement of the clinical outcomes, quality of life, Hs-CRP, and ESR in patients with mild-to-moderate UC.	[125]

**Table 4:** Application of curcumin in vivo-animal models.

Curcumin Form	Activity	Animal Model	Reference
Curcumin Nanocurcumin	Antidepressive effect Wound-healing Agent	Sprague–Dawley rats Male Wistar rats	[126] [127]
Curcumin, nanoparticles	Antibacterial and anti-inflammatory agent	Male C57BL/6 mice	[128]
Curcumin	Inhibitors of NF-κB	Mus musculus, C57BL/6J	[129]
Curcumin	Decontaminate and accelerate the Wound contraction	Wistar Rats	[130]
Curcumin, Nanoparticles	Adjuvant agent for the treatment of Hodgkin’s Lymphoma	Mice	[131]
Curcumin, Nanoparticles	Contrasting agent	Sprague–Dawley rats	[132]
Curcumin C3 Complex	Cancer prevention	Male C57BL/6 wild-type mice	[133]
Curcumin, Hydrogel	Wound-healing agent	Mus musculus var. albino mice	[134]
PVA/Chitosan/Curcumin Patches	Wound-healing agent	Wistar Rats	[135]

**Table 5:** Minimum inhibitory concentrations (MIC) of curcumin and fractional inhibitory concentration indices (FICIs) for potentially important pathogens of burn wounds [141].

Isolate	Genes	Curcumin MIC µg/mL	FICI
*Klebsiella pneumonie*	DHA	128	0.5
*Pseudomonas aeruginosa*	VEB	128	0.5
*Acinetobacter baumanni*	OXA-23, OXA-24	128	0.37
*Acinetobacter baumanni*	OXA-23, OXA-24	128	1
*Pseudomonas aeruginosa*	IMP-1	128	1
*Enterococcus faecalis ATCC 29212*	Type strain	128	0.26
*Pseudomonas aeruginosa*	GES	128	0.75
*Acinetobacter baumanni*	OXA-23, OXA-24	512	0.25
*Acinetobacter baumanni ATCC19606*	Type Strain	512	0.5
*Acinetobacter baumanni*	OXA-23, OXA-24	512	0.25
*Pseudomonas aeruginosa*	IMP-1	512	0.064
*Pseudomonas aeruginosa*	VIM-1	512	0.064
*Escherichia coli ATCC 25922*	Type Strain	256	0.4
*Klebsiella pneumonie ATCC700603*	Type Strain	256	0.5
*Klebsiella pneumonie*	NDM-6	256	0.28
*Klebsiella pneumonie*	NDM-1	256	0.56
*Klebsiella pneumonie*	NDM-6	256	0.56
*Pseudomonas aeruginosa*	IMP-2	256	0.56

**Table 6:** Antiviral, antiparasitic and antimalarial activity of curcumin.

Activity	Substance	Type of Microorganism	Therapeutic Effect	Reference
Antiviral				
	Curcumin, nanomicelles	Hepatitis C virus	The antiviral effects of curcumin nanomicelles on hepatitis C virus.	[167]
	Curcumin	Vesicular stomatitis virus	Determination of curcumin effects on vesicular stomatitis virus Dicer-1 expression.	[168]
	Curcumin	Chikungunya virus, zika virus	Antiviral activity of curcumin against zika and chikungunya virus.	[147]
	Curcumin, nanoparticles	Human immunodeficiency virus 1 (HIV-1)	Immunomodulatory activities of curcumin-stabilized silver nanoparticles on HIV-1.	[169]
	Curcumin	Enterovirus 71 (EV71)	Antiviral effects of curcumin on EV71.	[170]
	Curcumin	Human T lymphotropic virus 1 (HTLV-1)	Determination of curcumin on the expression of c-FLIP in HTLV-1-associated myelopathy/tropical spastic paraparesis (HAM/TSP) patients.	[171]
	Curcumin	Kaposi’s sarcoma-associated herpesvirus (KSHV or HHV8)	Antiviral activity of curcumin against KSHV replication and pathogenesis.	[146]
	Curcumin	Human immunodeficiency virus 1 (HIV-1)	Multifunctional mesoporous curcumin encapsulated iron phenanthroline Nanocluster on HIV-1.	[172]
	Curcumin	Zika virus	Inhibitory effects of novel natural products against zika virus.	[173]
	Curcumin, nanocurcumin	Dengue virus	Antiviral activity of curcumin encapsulated in nanoemulsion against dengue virus serotypes.	[174]
	Curcumin	Transmissible gastroenteritis virus	Antiviral effects of curcumin on transmissible gastroenteritis virus.	[175]
	Curcumin	Human parainfluenza virus type 3	Evaluation of curcumin on replication of human parainfluenza virus type 3.	[176]
	Curcumin	Hepatitis B virus	Evaluation of curcumin on viral entry of hepatitis B.	[156]
Antiparasitic and Antimalarial				
	Curcumin and netilmicin	*Leishmania major, Leishmania donovani*	Antileishmanial activity of netilmicin combined with curcumin significantly enhanced compared with when used alone.	[177]
	Nanoformulation of curcumin and miltefosine	*Leishmania donovani*	Combination therapy of curcumin with miltefosine exhibited a synergistic effect on both promastigotes and amastigotes under in vitro conditions.	[166]
	Curcumin Encapsulated to PLGA	*Plasmodium berghei*	Encapsulation of curcumin in PLGA led to increased parasite suppression about 56.8% at 5 mg/kg of nanoformulation, which was higher than in free curcumin (40.5%) at 10 mg/kg.	[178]
	Curcumin alone	*Giardia lamblia*	Curcumin inhibited giardia proliferation disrupted the cytoskeletal structures of trophozoites in the dose-dependent mode.	[160]
	Curcumin alone	*Fasciola gigantica*	A significant decrease was observed in the expression of glutathione-S-transferase and superoxide dismutase.	[179]
	Curcumin alone	*Cryptosporidium parvum*	The anticryptosporidial and antioxidant activity of curcumin against C. parvum were confirmed.	[180]
	Nanotized curcumin- benzothiophene conjugate	*Plasmodium falciparum*	The improved oral bioavailability of the nanotized formulation lowered the dosage at which the pharmacological effect was achieved while avoiding any observable adverse side effects.	[181]
	Curcumin, nanocomposite	*Plasmodium falciparum*	The antiparasitic effect of the nanocomposite on the metabolites of plasmodium falciparum	[182]

**Table 7:** Biological activities of curcumin.

Activity	Substance	Target	Therapeutic Effect	Reference
Anti-inflammatory				
	Curcumin	COX-2 NF-κB p-IκB ROS	Attenuates colistin-induced neurotoxicity in N2a cells via anti-inflammatory activity, suppression of oxidative stress, and a apoptosis.	[206]
	Curcumin	NF-κB COX-2	Attenuates airway inflammation and airway remoulding in cigarette smoke-induced COPD mice.	[207]
	Curcumin and rutin	COX-2	Reduce tumour-associated inflammation in HPV16-transgenic mice.	[189]
	Curcumin, curcumin and capsaicin	COX-2 IL-6 TGF-β	Combined curcumin and capsaicin are efficient against the lipopolysaccharide Induced expression of proinflammatory cytokines in peripheral blood mononuclear cells.	[208]
Antioxidant				
	Curcumin-loaded sodium alginate/ZnO hydrogel beads	DPPH Assay	Composite hydrogel beads have protected curcumin from light degradation can therefore prolong its antioxidant activity.	[209]
	Curcumin	MDA SOD GSH-Px	Curcumin protects the liver, kidneys and brain from the oxidative damage caused by irradiation.	[210]
	Curcumin, curcumin and piperine	MDA SOD Catalase	Curcumin may be used as an adjunct therapy in individuals with oxidative stress.	[211]
	Curcumin	MDA total Antioxidant Capacity (TAC)	Pure curcumin reduces MDA concentration and increases total antioxidant capacity.	[212]
Antinociceptive				
	Curcumin	DRG Neurons β-Endorphin and Enkephalin	The curcumin attenuates cancer-induced bone pain	[213]
	Curcumin	γ-Aminobutyric Acid (GABA) and Opioid Receptors	Antinociception of curcumin	[214]
	Curcumin-loaded PLGA nanovesicles (PLGA-CUR)	Cytokine and BDNF	Antinociceptive effects of PLGA-CUR	[201]
	Curcumin	The acid-Sensing Ion Channels (ASICs)	Antinociceptive Effects of Curcumin	[215]
Wound healing agent				
	PVA/chitosan/curcumin patches	Cell Line Studies and MTT Assay	Antibacterial activity of PVA/Chi/Cur against four major bacterial strains commonly found in wound sites and water retainability indicates it to be a perfect material for wound treatment.	[135]
	Nanocurcumin	Fibroblast, Collagen, Reepithelization	Curcumin nanoformulation enhanced wound repair by inhibiting the inflammatory response, stimulating angiogenesis, inducing fibroblast proliferation and enhancing reepithelization and synthesis of collagen.	[127]
	Curcumin, Hydrogel	L929 Fibroblast Cells	Curcumin incorporation accelerates full-thickness skin wound healing	[114]

## Data Availability

Data available in a publicly accessible repository.
